# Medium- and long-term effects of endovascular treatments for severely stenotic basilar arteries supported by multimodal imaging

**DOI:** 10.1186/s12883-020-01863-5

**Published:** 2020-07-31

**Authors:** Yuanzhi Li, Zhenfa Li, Ligang Song, Weimin Xie, Xianghao Gong, Dongliang He, Xin Zhang

**Affiliations:** 1 Department of Neurosurgery, Affiliated Hengyang Hospital, Southern Medical University (Hengyang Central Hospital), Hengyang, 421001 China; 2 Department of Vascular Surgery, Affiliated Hengyang Hospital, Southern Medical University (Hengyang Central Hospital), 12# Yancheng road, Hengyang, 421001 Hunan province China; 3 Department of Neurointervention, Affiliated Tiantan Hospital, Capital Medical University, 119# west south fourth ring road, fengtai district, Beijing, 100050 China; 4 Department of Gynecology, Affiliated Hengyang Hospital, Southern Medical University (Hengyang Central Hospital), Hengyang, 421001 China; 5 Department of Science and Education, Affiliated Hengyang Hospital, Southern Medical University (Hengyang Central Hospital), Hengyang, 421001 China; 6 Department of Nutrition, Affiliated Hengyang Hospital, Southern Medical University (Hengyang Central Hospital), Hengyang, 421001 China; 7 Department of Neurosurgery, Zhujiang Hospital, Southern Medical University, Guangzhou, 510282 China

**Keywords:** Basilar artery, Stenosis, Atherosclerosis, Stent implantation, Angioplasty

## Abstract

**Background:**

To evaluate the medium-and long-term effect of intravascular interventional therapy for symptomatic severe basilar artery stenosis supported by multimodal imaging.

**Method:**

After strict screening of 67 patients with symptomatic severe basilar artery stenosis (70–99%) with atherosclerotic stenosis, 67 patients with symptomatic recurrence after intensive drug treatment were treated with intravascular balloon dilatation and Enterprise stent implantation. Any stroke or death within 30 days after operation and any stroke and restenosis during medium-and long-term follow-up were recorded.

**Results:**

①The mean age of 67 patients (67lesions) was 57 ± 8 years old, and the technical success rate was 100%; ②Preoperative angiography showed that the collateral circulation was poor, and TICI was 1-2a while postoperative angiography showed that TICI was significantly improved to 2b-3; ③The average preoperative stenosis rate was 82 ± 9%, and the postoperative stenosis rate was reduced to 17 ± 10%; ④Before surgery, abnormal perfusion was found in the posterior circulation CTP; After the postoperative re-examination, the posterior circulation of CTP perfusion was significantly improved; ⑤Postoperative symptoms and neurological conditions improved significantly; ⑥Complications of perforating branch event occurred in 1 case after operation, and symptoms were relieved after more than 1 month of medication treatment, and mild neurological dysfunction remained. 1 case developed subacute thrombosis in the stent, which improved after active intra-arterial thrombolysis, and there was no residual neurological dysfunction; and 1 case of micro-guide wire being trapped by the distal vasospasm. ⑦67 patients were followed up by telephone, WeChat or imaging for 36–66 months.

**Conclusions:**

In summary intravascular balloon dilation + Enterprise stent implantation is safe and effective for the treatment of symptomatic severe atherosclerotic stenosis of the basilar artery, with high technical success rate, low perioperative complications, and good mid-term and long-term effects.

## Background

Stroke is one of the leading causes of disability and death in adults. Intracranial arterial stenosis is the narrowing of the main intracranial arteries due to the formation of atherosclerotic plaques, which may represent the most common cause of stroke worldwide. Intracranial arterial stenosis is more common in Asians, particularly in the Chinese population. Among all stroke populations in China, The incidence and prevalence of ischemic stroke are as high as 69.6 and 77.8%, respectively [1], and have been increasing annually and are more commonly observed in younger populations [2]. More than 40% of ischemic stroke cases are related to compromised blood circulation of the vertebrobasilar artery [3]. According to the Chinese Intracranial Atherosclerosis (CICAS) study [4], the prevalence of intracranial atherosclerotic stenosis (ICAS) is as high as 46.6% in the Chinese population of stroke patients, and the basilar artery is the most vulnerable component of the affected blood vessels. Even following adherence to current standard medications, patients still exhibit high morbidity. The risk of disease-related ischemic stroke is especially high in symptomatic patients with a high degree of atherosclerotic stenosis (> 70%) [5]. Therefore, development of effective interventions to prevent stroke in patients with ICAS is necessary, especially for those with symptomatic ICAS. To this aim, leveraging technological advancements and updated materials may facilitate development of safer and more efficacious endovascular stent implantations for the treatment of symptomatic severe basilar artery stenosis. The present study screened 67 patients with symptomatic atherosclerotic stenosis in the basilar arteries and evaluated the safety, as well as the medium- and long-term effects, of endovascular interventions with an Enterprise stent system.

## Methods

### Research subjects

Sixty-seven patients with severe atherosclerotic stenosis in the basilar arteries who were admitted and treated with Enterprise stents at Tiantan hospital, Hengyang central hospital and Zhujiang hospital from April 2014 to April 2016 were continuously screened. All patients signed written informed consents before participating in the study, and the research protocol of this study was approved by the Ethics committee of the hospital. Inclusion criteria for patients in the present study were as follows: (1) 18–80 years old; (2) 70–99% extent of stenosis with the length of stenosis being less than 15 mm, as measured based on the standard vertebral method established by the Warfarin–Aspirin Symptomatic Intracranial Disease (WASID) trial [6], using the same image magnification in digital subtraction angiography (DSA); (3) standard medical drug treatment for 1–3 months and non-disabling posterior circulation infarct (PCI) and transient ischemic attack (TIA) recurrence in the area receiving blood supply from the affected vessels; and (4) the last episode of symptoms occurring at least 3 weeks before the scheduled operation. Exclusion criteria for patients in the present study were as follows: (1) presence of non-atherosclerotic stenosis; (2) symptoms of perforating stroke; (3) potential cardiac-derived embolism, intracranial hemorrhage within 3 months, intracranial tumors, aneurysms, venous malformation, or moyamoya disease; or (4) abnormal blood coagulation, clopidogrel resistance, or presence of heparin, aspirin, or clopidogrel contraindications.

### Preoperative assessments

All patients were preoperatively assessed by multimodal imaging, including transcranial Doppler sonography (TCD), computed tomography angiography (CTA), computed tomography perfusion (CTP), magnetic resonance angiography (MRA), diffusion weighted imaging (DWI), high-resolution magnetic resonance imaging (HR-MRI), perfusion-weighted imaging (PWI), and digital subtraction angiography (DSA) of the entire cerebrovascular system. Measurement of stenosis was performed according to the standard vertebral method established by the WASID. The DSA images at the same magnification were used to measure the degree of stenosis, the length of stenosis, the conditions of proximal and distal lesions, the relationship between branch vessels and plaques, and the collateral circulation and forward blood flow conditions of the patients. CTP or PWI was used to evaluate cerebral perfusion; HR-MRI was used to determine the eccentricity and concentricity of the plaque.

### Surgical procedures

**Fig. 1:**
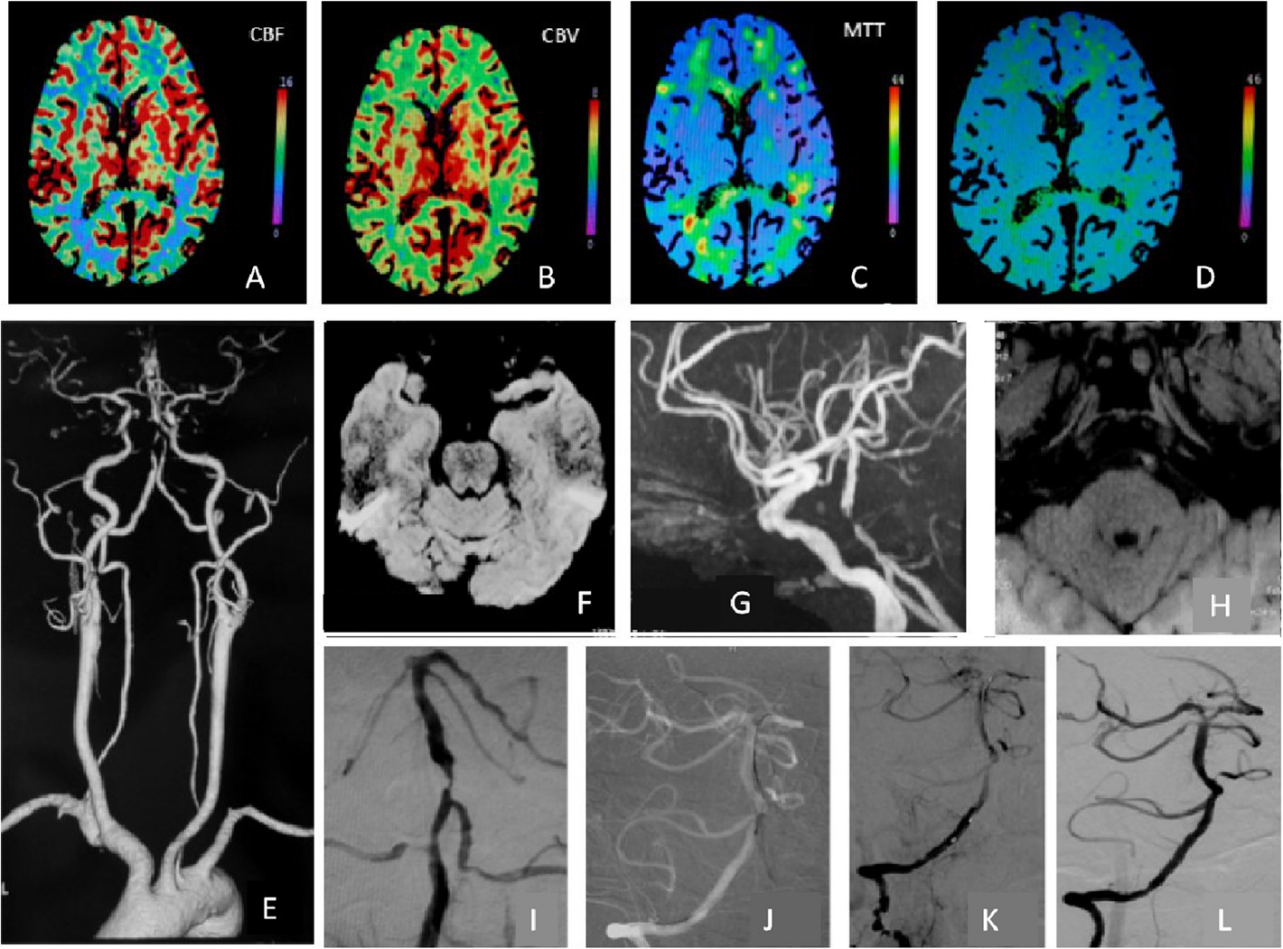
Multimodal imaging assessment and surgical procedures. **a**-**d** Preoperative, posterior circulation CTP perfusion was abnormal. **e** CTA of the head and neck demonstrates severe stenosis of the lower basilar artery. (-**h** DWI images showed no acute cerebral infarction, MRA images show severe stenosis in the lower basilar artery, HR-MRI images show basilar eccentric stenosis. **i** Before the operation, DSA images showed severe stenosis in the lower basilar artery. **j**-**k** During the procedure, balloon expansion and stent implantation were performed. **l** Postoperative, DSA images showed significant improvement in severe basilar stenosis

All operations in this study were performed under general anesthesia. Systemic heparinization was induced in all patients throughout the surgical procedures. A 6F or 5F Envoy guiding catheter (Cordis, Santa Clara, CA) was placed into the distal segment of the V2 vertebral artery at a level of approximately C4–C2 of the cervical spine (depending on the diameter of the target vertebral artery). A transradial approach was used instead of a transfemoral approach if there was difficulty in reaching the target vessel. A dosage of 70 U of heparin/kg bodyweight was injected after the completion of intubation. The degree of stenosis was calculated based on the diameter of the normal vessel (WASID standard) near the distal end of the lesion, and was either calibrated with a contrast-filled catheter as a reference or was measured by a DSA workstation. All lesions were pre-expanded with a Gateway balloon (Boston, MA), followed by implantation with an Enterprise stent (Cordis). According to our experience, the selection of the Gateway balloon was comprehensively based on the distribution of the adjacent perforating branch of the lesion, the degree of stenosis, the length of the lesion, the degree of angulation of the lesion, the location and texture of the plague, and 70–80% of the normal blood vessel diameter at the distal end of the lesion. In addition, multiple expansions of the balloon were avoided. Patients with preoperative assessments of hard plaque textures were subjected to extended balloon expansions lasting 3 min under the corresponding pressure until achieving sub-satisfactory results of the lesion angioplasty. Stent selection was based on both ends of the stent exceeding the lesion by 3–5 mm. Although the Enterprise stent could be recycled and released repeatedly, every effort was made for its accurate placement in the first release to avoid multiple releases and cutting of the plaque. After the stent release was completed, a vertebral arteriography was performed immediately, and the residual stenosis was measured. The stenosis was observed simultaneously for 10 min, and an angiography was performed to determine the forward blood flow. In addition to the intraoperative heparinization, nimodipine was used to prevent cerebral vasospasms and to control systolic blood pressure to reside between 110 and 130 mmHg. The criteria for successful stent implantation consisted of complete coverage of the lesion by the stent, no drift of the stent, no new neurological deficits, and < 50% residual stenosis (Fig. 1).

### Perioperative management

All patients were subjected to risk-factor control and improvement of living habits. Patients were also orally administered aspirin (100 mg/day) and clopidogrel (75 mg/day) for more than five consecutive days before the operation. Patients who had poor thromboelastographies after antiplatelet aggregation medication were subsequently treated with antiplatelet drugs.

Intravenous nimodipine (4.2 ml/h) was given continuously for 2 h before the operation to prevent cerebral vasospasms. A complete skull CT immediately after surgery was used to confirm no intracranial hemorrhage. Each patient was given moderate sedation in the neurological intensive care unit and a subcutaneous injection of low-molecular-weight heparin once every 12 h for two to 3 days. After the stent implantation, each patient was given 100 mg/day of aspirin and 75 mg/day of clopidogrel for 90 days, followed by continued administration of 100 mg/day of aspirin for life. For postoperative risk-factor control, systolic blood pressure and low-density lipoproteins of the patients were controlled under 130 mmHg (< 120 mmHg for diabetic patients) and 70 mg/dl (1.81 mmol/l), respectively, or low-density lipoproteins were reduced by 50%. Patients were asked to quit smoking, change their eating habits, lose weight, avoid being sedentary, and exercise properly.

### Follow-ups

Patients were postoperatively followed up by telephone, WeChat, or imaging review. The assessments were performed by the same neurologist who performed the surgery, with PCI, TIA, and incidence of death being monitored for 30 days. The National Institute of Health stroke scale (NIHSS) was used for assessments of patients before admission and at discharge. During the clinical follow-ups, patients who were suspected to have recurrent stroke were subjected to head CT or MRI examinations. Patients with stent implantations were followed up 3 months after being discharged, and their TCD/CTA, MRA, or DSA were reviewed after 6–12 months. Patients who were asymptomatic were followed up every 6 months. The success rate of stent implantation, incidence of perioperative complications, degree and rate of restenosis, speed of stenotic blood flow, and incidence of ischemic events of all patients were recorded. Restenosis was considered as the post-stent implantation arterial diameter being less than 50% of the immediate arterial diameter after the implantation.

### Statistical analysis

All statistical analysis was performed using SPSS 22.0 software (IBM SPSS Inc., Chicago, IL). Data are presented as the mean ± standard deviation (x ± s). Comparisons between two distributions were performed using *t*-tests or Wilcoxon. Comparisons among more than two distributions were performed by one-way analyses of variance (ANOVAs). Categorical counted data were compared using chi-square (χ^2^) tests. A *P* < 0.05 was considered statistically significant.

## Results

### Results after stent implantations

A total of 67 patients (57 ± 8 years old), including 57 males and 10 females, were included in the present study. Symptoms lasted from 3 weeks to 90 days after the stent implantations. A total of 67 Enterprise stents were implanted into the patients, with a 100% technical success rate. The average preoperative stenosis rate was 82 ± 9%, and the postoperative stenosis rate was reduced to 17 ± 10%. There were 38 cases of ≤5-cm lesion lengths, 18 cases of 5–10-cm lesion lengths, and 11 cases of 11–15-cm lesion lengths. The average stenosis length was 7 ± 4 mm. Before the stent implantation, there were eight cases of TICI grade 1 (11.9%), 47 cases of TICI grade 2a (70.2%), and 12 cases of TICI grade 2b (17.9%); after the stent implantation, there were 37 cases of TICI grade 2b (55.2%) and 30 cases of TICI grade 3 (44.8%) (Table 1). Comparison of pre- and postoperative results revealed a significant improvement of forward blood flow (Χ^2^ = 97.755, P<0.001). The peak blood flow velocity in the basilar artery stenosis monitored by TCD was significantly decreased from 226 ± 21 cm/s preoperatively to 127 ± 13 cm/s postoperatively (t = 21.39, *P* < 0.001). The preoperative and postoperative neurological functions assessed by NIHSS scores were 1.067 ± 0.122 and 1.005 ± 0.108, respectively, and this difference was statistically significant (Z = − 3.178, *P* < 0.01). Comparison of preoperative and postoperative CTP parameters of the circulating blood supply area (Table 2) showed no significant difference in cerebral blood volume (CBV, *P* > 0.05), whereas there were a significant differences in cerebral blood flow (CBF, increase in CBF postoperatively, *P* < 0.05), postoperative time to peak (TTP, decrease in TTP postoperatively, *P* < 0.001), and mean transit time (MTT, decrease in MTT postoperatively, *P* < 0.01).

**Table 1:** Comparison of preoperative and postoperative TICI

TICI	1	2a	2b	3
Preoperative	8	47	12	0
Postoperative	0	0	37	30

postoperative vs. preoperative, Χ^2^ = 97.76, *P* = 0.000

**Table 2:** Comparison of preoperative and postoperative CTP (CBF, ml·100 g^− 1^·min^− 1^; CBV, ml·100 g^− 1^; TTP, s; MTT, s)

Time	CBF	CBV	TTP	MTT
Preoperative	52.86 ± 9.03	30.78 ± 4.39	13.12 ± 2.59	7.11 ± 1.48
Postoperative	59.67 ± 8.14	31.99 ± 3.71	10.85 ± 2.31	4.75 ± 0.81
T-value	−2.422	0.159	6.667	1.621
*P*-value	0.023	0.811	0	0.014

### Results of postoperative complications

**Fig. 2:**
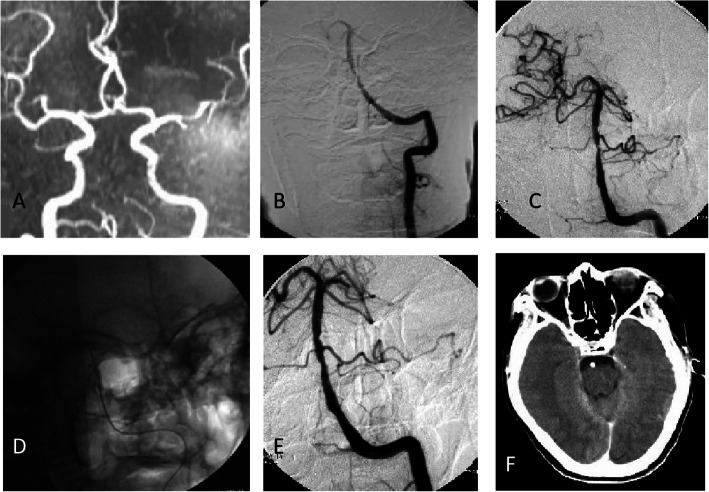
Management of subacute thrombosis in the stent. **a** Preoperative MRA images showed subacute occlusion after basilar stenting. **b** Preoperative DSA images showed acute occlusion after basilar stenting. **c**-**d** Intraoperative, intra-arterial thrombolysis and balloon dilation. **e** Postoperative, subacute occlusion of basilar artery was recanalized. **f** Postoperative CT images showed no intracranial hemorrhage

Among the 67 patients, three patients had complications within 30 days after the surgery, including the following: one case (1.5%) of complications of a perforating branch event, for which the symptoms of this patient were relieved after active medication treatment for more than a month, whereas mild residual neurological dysfunction remained; one case (1.5%) of subacute thrombosis development in the stent, which was improved after active intra-arterial thrombolysis, and no residual neurological dysfunction was found (Fig. 2); and one case of microwire being clamped by the distal vasospasm. This last patient was treated with antivasospasm medication, and the microwire was pulled out the next day after the surgery, after which no residual neurological dysfunction was found. The overall rate of perioperative complications was 4.5% (3/67). No new PCI or TIA occurred in the area receiving blood supply from the offending vessels, and no hyper-perfusion, arterial dissection, or death occurred.

### Follow-up results

Sixty-seven patients who were followed up by telephone, Wechat, or imaging re-examination had an average of 56 ± 21 months (36–66 months) of follow-ups, including 55 cases (82%) that included regular imaging reviews and 12 cases of phone or Wechat follow-ups (the latter 12 patients refused to have imaging-review follow-ups). Four patients (95% confidence interval [CI] 0.866–0.998) exhibited stenosis in the stent, and three of these four patients had stable symptoms. Continued imaging-review follow-ups showed that one patient had TIA and was consequently treated with balloon dilation. The remaining patients did not have TIA or PCI in the area receiving blood supply from the offending vessels during follow-ups.

## Discussion

ICAS is one of the most common causes of ischemic stroke and has a high risk of recurrence, especially in Asian populations [4, 7]. More than 40% of ischemic stroke cases are related to impaired blood circulation of the vertebral and basilar arteries. The risk of disease-related ischemic stroke is higher in symptomatic patients with a high-degree of atherosclerotic stenosis (> 70%) [8]. Patients with ischemic stroke in the posterior circulation usually have more severe symptoms than those with ischemic stroke in the anterior circulation [9]. For ICAS, a number of randomized controlled trials have been conducted to explore the best treatment strategies for preventing the recurrence of ischemic stroke [10–12]. However, the results suggest that intensive drug therapy combined with the control of risk factors has been clearly more efficacious than that of intracranial stenting, which is mainly due to the low recurrence rate of stroke after drug therapy and the relatively high incidence of complications related to endovascular therapy. As such, it may be difficult to perform more efficacious intravascular treatment of ICAS compared to that of drug therapy. However, according to the SAMMPRIS study, patients who received intensive medication had a 5.8% primary-endpoint event rate within 30-day follow-ups, 12.6% one-year endpoint event rate, and a 14.9% three-year endpoint-event rate. In the VISSIT trial, patients who received intensive medication had an even higher 30-day primary-endpoint event rate and one-year endpoint event rate compared to those reported from the SAMMPRIS study. Although the incidence of endpoint events in patients receiving intensive medication is lower than that of patients who receive intracranial stenting, the incidence of endpoint events is still high and unacceptable. The adverse events in these studies occurred after Winspan stenting, suggesting that such adverse events may be related to the open-loop design, large radial force, high head-end hardness, and severe tortuosity of Wingspan stents that make them difficult to pass through blood vessels; in addition, the Wingspan stent is difficult to operate and can easily induce perforating branch events [13]. Therefore, based on our experience in the treatment intracranial aneurysms, we chose Enterprise stents in our present study, which is a more mature stent for ICAS treatment. In addition, after the above two major studies, the CASSISS trial held in China released the first-stage results in 2015 [14]. The CASSISS trial continuously included 100 patients and implanted Winspan stents in 13 large centers, which showed a success rate of stent implantation of 100% and a 2% incidence of stroke and death in the postoperative 30 days, which was significantly lower than the 14.7 and 24.1% in the SAMMPRIS and the VISSIT trials, respectively. These results provide hope for further exploring ICAS endovascular treatments.

The Enterprise stent is a closed-loop self-expanding stent for assisting spring-coil embolization of wide-necked intracranial aneurysms, with a stent diameter of 4.5 mm and which consists of four different lengths—14, 22, 28, and 37 mm—that are suitable for 2.5–4.0-mm intracranial blood vessels. The Enterprise stent has a release of less than 70% and is recyclable. It has a closed-loop design so that it is easily passed through curved blood vessels, and its radial support force is smaller than that of the Winspan stent. The operation and release of the Enterprise stent are simpler than those of the Winspan stent, and its positioning is comparatively more accurate. Moreover, the Winspan stent is more prone to cause intimal hyperplasia and irritation to the vessel wall. Not many Enterprise stenting reports for ICAS treatment are currently available. A study by Vajda et al. [15] used the Enterprise stent for the treatment of symptomatic ICAS, with a success rate of 100%. The main complications occurred in four cases (9.1%) during the perioperative period (30 days), including three cases (6.8%) of ischemic perforating stroke and one case (2.2%) of reperfusion stroke. Among the 42 patients with a median follow-up of 25.6 months (range of 12–57 months), no further TIA or stroke was found. Among the 38 patients who underwent imaging-review follow-ups, three cases (6.81%) experienced > 50% restenosis in the stent after an average follow-up of 22 months. Our present study showed that for patients with symptomatic ICAs, applications of small-sized balloon angioplasty and the Enterprise stent implantation were technically successful with a lower incidence of complications. A study by Feng et al. [16] used the Enterprise stent for symptomatic ICAS treatment, which yielded a successful operation rate of 100% and the stenosis decreased from 79.3 ± 8.1% to 14.9 ± 12.3%. The perioperative ischemic stroke rate was 6.8%, and the hemorrhagic stroke rate was 2.2%. There were no further TIA or strokes during an average follow-up of 25.6 months, and restenosis in the stent was > 50% in 6.8% of the cases. The promising results of these two studies offer hope for improving ICAS treatments. However, these two studies performed ICAS stent implantations in the anterior circulation. Although they had high successful operation rates and the incidences of short-term and long-term postoperative complications were lower than those in the SAMMPRIS trial, they did not perform detailed subgroup analysis, and the number of cases in the basilar artery group was relatively small. In addition, the specific perioperative complications, long-term results, and restenosis of the basilar artery subgroup were unclear. To further evaluate the long-term effect of Enterprise stents in the treatment of severe basilar artery stenosis, our present study performed long-term follow-ups and ultimately yielded more efficacious results. Two cases were found to have complications within the 30-day postoperative follow-ups, and one case (2.7%) had complications from perforating branch events. After active treatment, the symptoms of this patient were relieved for more than a month, and mild residual neurological dysfunction was found. Another case (2.7%) with postoperative complications developed subacute thrombosis in the stent, which was improved after active intra-arterial thrombolysis. Subsequently, no neurological dysfunction remained, with an overall perioperative rate of 5.4% (2/37). No new PCI or TIA occurred in the area receiving blood supply from the offending vessels during follow-ups, and no hyper-perfusion, arterial dissection, or death occurred. For patients who were considered to only have neurological dysfunction as a complication, their overall perioperative complications were 2.7%. The mean follow-up period was 56 ± 21 months (36–66 months). Intra-stent stenosis occurred in two patients (5.4%), and one of them had stable symptoms and continued imaging-review follow-ups. This patient had subacute thrombosis. One patient had TIA and underwent balloon dilation. The remaining patient had no TIA or PCI in the area receiving blood supply from the offending vessels during follow-up and they had 2.7% actual symptomatic stenosis. Collectively, the results of these studies were more promising than those of the restenosis rates of the Wingspan stents (31.2%).

In 2019, the long-awaited WEAVE trial was published [17], which confirmed the perioperative safety of the Wingspan stent. In this trial, the perioperative stroke and mortality rate was 2.6%, which was better than the set target of a 4% event incidence. However, the long-term results of the WEAVE trial require further follow-up. Comparing our results with the WEAVE trial showed that although perioperative complications occurred in two of our cases, basilar artery stenosis accounted for 14% (22 cases) of cases in the WEAVE trial, including two non-fatal strokes, two deaths from stroke, and a total of four patients who had an index event of stroke, hemorrhage, or death. Of the two non-fatal stroke cases, one patient had stroke in the pontine perforating branch of the basilar artery, with the postoperative modified Rankin Scale (mRS) being reduced to a score of 4. Although the incidence of stroke due to perforator occlusion was only 0.7% (1/152) and the total number of perforator events was low, the events affecting the perforating branches of the basilar artery in the WEAVE trial likely reached 4.5% (1/22), which was higher than that of our present findings. Thus, we conclude that Enterprise stenting is more beneficial in the treatment of basilar artery stenosis compared to that of Wingspan stenting. Further follow-ups of the WEAVE trial are needed because this study did not analyze the long-term efficacy of stenting and restenosis.

Our present study focused on preoperative assessment, perioperative management, surgical timing, surgical experience, perforator protection, and problems of restenosis. We used multimodal imaging to evaluate the lesion length, degree of stenosis, cerebral perfusion, and collateral circulation. Additionally, our multimodal imaging was supplemented with HR-MRI to monitor plaque location, shape, signal, thickness, and presence of localized or circular plagues. We also measured the nearby parenchymal signal as a reference to determine the plague texture, and the plague signals were categorized into equal signal, slightly lower/low signals, slightly higher/high signals, and mixed signals. HR-MRI has also been used to analyze plaque textures and locations to minimize events affecting the perforating branches of the basilar artery, especially for patients with hyperlipidemia and hyperglycemia [18]. In addition, multimodal imaging assessment has also been shown to be beneficial for preventing hyper-perfusion [19]. In the present study, during the perioperative period, we controlled blood pressure at a relatively low level of 120–130 mmHg, and we used the implementation of appropriate sedation to reduce the occurrence of cerebral hemorrhage and hyper-perfusion [20]. The bowstring effect should be avoided when loosening the balloon and stent system intraoperatively to prevent tearing of the perforating vessels and to achieve sub-satisfactory results once the balloon is expanded [21]. Perforator stroke (PS) is one of the most common complications of intracranial angioplasty and/or stent implantation [13]. The basilar artery is the most common site of PS among all perforator-rich areas. In the SAMMPRIS trial, the incidence of PS in the basilar artery was 16%. The high perioperative PS rate and its unique blood supply through perforating branches make the basilar artery a key location for studying such complications. Diabetes was associated with a significantly higher incidence of PS (11.4% vs. 2.3%, *P* = 0.004). Dyslipidemia was another risk factor for atherosclerosis and is associated with a high incidence of PS (7.6% vs. 1.8%, *P* = 0.045). The incidence of PS in patients with < 18 days difference between the time of the last symptom to the time of operation was higher than that of the patients with > 18 days difference (8.9% vs. 1.5%, *P* = 0.009), and 5.1% of patients were found to be associated with the PS related to surgery. Therefore, we adjusted our surgical timing to be 3 weeks after the last symptom onset [22]. The main cause of restenosis is the development of intimal hyperplasia, which might be related to dilated trauma, stent thrombosis, inflammatory responses of the blood vessel wall to the stent, and high radial force stimulation. The prevention of restenosis in our present study was likely due to reducing the number of balloon expansions, performance of sub-satisfactory expansion, and our choice of the Enterprise stent. Although the Enterprise stent has the capacity of recovery and release, evert effort was made to accurately localize the stent and to complete the release in one attempt. Moreover, the characteristics of the Enterprise stent also reduced the stimulation and cutting of the plague.

## Conclusions

In summary, patients who underwent endovascular treatments were evaluated via multimodal imaging in the present study. Intravascular balloon dilation and angioplasty combined with Enterprise stent implantation was found to be a safe and effective technique for treating severe atherosclerotic stenosis of the basilar artery, which yielded a high success rate, low perioperative complication rate, and achieved efficacious medium- and long-term therapeutic outcomes.

## Data Availability

The datasets used and/or analyzed during the current study are available from the corresponding author on reasonable request.

## Abbreviations

CICAS: Chinese intracranial atherosclerosis study
ICAS: Intracranial atherosclerotic stenosis
WASID: Warfarin–aspirin symptomatic intracranial disease trial
PCI: Posterior circulation infarct
TIA: Transient ischemic attack
HR-MRI: High-resolution magnetic resonance imaging
SAMMPRIS: Stenting versus aggressive medical therapy for intracranial arterial stenosis
CASSISS: China angioplasty and stenting for symptomatic intracranial severe stenosis
VISSIT: The vitesse stent ischemic therapy
WEAVE: Wingspan stent system post market surveillance
